# Comparative Analysis of the Complete Chloroplast Genomes of Five *Quercus* Species

**DOI:** 10.3389/fpls.2016.00959

**Published:** 2016-06-28

**Authors:** Yanci Yang, Tao Zhou, Dong Duan, Jia Yang, Li Feng, Guifang Zhao

**Affiliations:** Key Laboratory of Resource Biology and Biotechnology in Western China (Ministry of Education), College of Life Sciences, Northwest UniversityXi’an, China

**Keywords:** *Quercus*, chloroplast genome, repeat, nucleotide substitution, positive selection, plastid marker, phylogeny

## Abstract

*Quercus* is considered economically and ecologically one of the most important genera in the Northern Hemisphere. Oaks are taxonomically perplexing because of shared interspecific morphological traits and intraspecific morphological variation, which are mainly attributed to hybridization. Universal plastid markers cannot provide a sufficient number of variable sites to explore the phylogeny of this genus, and chloroplast genome-scale data have proven to be useful in resolving intractable phylogenetic relationships. In this study, the complete chloroplast genomes of four *Quercus* species were sequenced, and one published chloroplast genome of *Quercus baronii* was retrieved for comparative analyses. The five chloroplast genomes ranged from 161,072 bp (*Q. baronii*) to 161,237 bp (*Q. dolicholepis*) in length, and their gene organization and order, and GC content, were similar to those of other Fagaceae species. We analyzed nucleotide substitutions, indels, and repeats in the chloroplast genomes, and found 19 relatively highly variable regions that will potentially provide plastid markers for further taxonomic and phylogenetic studies within *Quercus*. We observed that four genes (*ndhA*, *ndhK*, *petA*, and *ycf1*) were subject to positive selection. The phylogenetic relationships of the *Quercus* species inferred from the chloroplast genomes obtained moderate-to-high support, indicating that chloroplast genome data may be useful in resolving relationships in this genus.

## Introduction

The genus *Quercus* (Fagaceae) is distributed throughout the Northern Hemisphere, and consists of approximately 500 species (Nixon, 1993; Manos et al., 1999). Oak taxonomy is perplexing, because of intermediate morphological traits caused by extensive hybridization (Rushton, 1993; Cavender-Bares et al., 2004; Curtu et al., 2007; Burgarella et al., 2009; Moran et al., 2012), introgression, incomplete lineage sorting, and convergent evolution (Kole, 2011). Based on pollen characteristics and nuclear markers, six major intrageneric groups (*Cyclobalanopsis*, *Cerris*, *Ilex*, *Lobatae*, *Protobalanus*, and *Quercus*) have been identified (Oh and Manos, 2008; Denk and Grimm, 2009, 2010; Hubert et al., 2014). In China, *Quercus* has generally been divided into five sections, based on morphological characteristics (Zhou et al., 1994; Pu et al., 2001; Peng et al., 2007). Among these, Sect. *Echinolepides* is an intermediate group between evergreen oaks (Sect. *Brachylepides* and Sect. *Engleriana*) and deciduous oaks (Sect. *Aegilops* and Sect. *Quercus*). However, the phylogenetic relationships among *Quercus* species are still not fully understood because of incomplete sampling, the use of markers with insufficient phylogenetic signals, and complex evolutionary issues.

Because of their highly conserved structure, general recombination-free, uniparental inheritance, and small effective population sizes (Birky et al., 1983), chloroplast (cp) DNA sequences have been extensively employed to resolve plant phylogenies (Jansen et al., 2007; Moore et al., 2010; Shaw et al., 2014). With the rapid development of next-generation sequencing, it is now cheaper and faster to obtain genomes than by traditional Sanger sequencing (Alkan et al., 2011). Therefore, cp genome-scale data have been increasingly used to infer phylogenetic relationships at high taxonomical levels, and even in lower taxa, great progress has been made (Jansen et al., 2007; Moore et al., 2007, 2010; Parks et al., 2009; Barrett et al., 2013; Ma et al., 2014; Carbonell-Caballero et al., 2015). Most angiosperm cp genomes have a quadripartite circular structure, and are composed of two copies of inverted repeat (IR) regions that are separated by a large single copy (LSC) region and a small single copy (SSC) region (Jansen et al., 2005; Jansen and Ruhlman, 2012). Despite the fact that angiosperm cp genomes exhibit a remarkably conserved gene content and order (Jansen and Ruhlman, 2012), some lineages (such as Campanulaceae, Fabaceae, Geraniaceae, and Oleaceae) exhibit different levels of genomic upheaval, such as gene, intron, or even IR region loss, gene duplications, and large-scale rearrangements (Cosner et al., 2004; Lee et al., 2007; Cai et al., 2008; Guisinger et al., 2010, 2011; Martin et al., 2014).

In the present study, the comparative analysis of five complete *Quercus* cp genomes was conducted in order to explore the sequences’ molecular evolution. Highly variable regions were identified that could serve as potential markers for phylogenetic analysis or candidate DNA barcoding in future studies.

## Materials and Methods

### Plant Material and DNA Extraction

The materials used were *Q. dolicholepis*, *Q. variabilis*, *Q. aliena*, and *Q. aliena* var. *acuteserrata*. Voucher specimens of these species were deposited in the herbarium of Northwest University, Xi’an, China. Total genomic DNA was isolated from silica-dried leaf material using a modified CTAB method (Doyle, 1987), which was conducted by Biomarker Technologies, Inc. (Beijing, China). The complete cp genome of *Quercus baronii* (GenBank accession No. KT963087; Yang et al., 2015) was recovered in order to conduct a comparative analysis with these four species.

### Illumina Sequencing, Assembly, and Annotation

Total genomic DNA was sequenced using an Illumina Hiseq 2500 platform by Biomarker Technologies, Inc. Firstly, all of the raw reads were trimmed using a CLC Genomics Workbench v7.5 (CLC Bio, Aarhus, Denmark) with the default parameters set. Reference-guided assembly was then used to reconstruct the chloroplast genomes with the program MITObim v1.7 (Hahn et al., 2013; **Table 1**). In this process, in order to obtain accurate sequences, every species was assembled five times with the reference genomes *Q. rubra* (JX970937), *Q. spinosa* (KM841421), *Q. aquifolioides* (KP340971), *Q. aliena* (KP301144), and *Castanea mollissima* (HQ336406). A few gaps in the assembled cp genomes were corrected by Sanger sequencing. Primers were designed using Lasergene 7.1 (DNASTAR, Madison, WI, USA). Primer synthesis, and the sequencing of the polymerase chain reaction products, was conducted by Sangon Biotech (Shanghai, China). The primers and amplifications are shown in Supplementary Table S1. The complete cp genomes were annotated using the program DOGMA (Wyman et al., 2004), and then manually corrected by comparing them with the complete cp genomes of the abovementioned, related species in GENEIOUS R8 (Biomatters, Ltd., Auckland, New Zealand). Circular plastid genome maps were drawn using OGDRAW^1^ (Lohse et al., 2013).

**Table 1:** Assembly information for the five *Quercus* species.

Species	Locality	GenBank numbers	Assembly reads	Mean coverage
*Quercus baronii*	Baoji, Shaanxi, China	KT963087	298,797	230x
*Q. aliena*	Xi’an, Shaanxi, China	KU240007	617,114	481x
*Q. aliena* var. *acuteserrata*	Maoxian, Sichuan, China	KU240008	560,430	435x
*Q. variabilis*	Xi’an, Shaanxi, China	KU240009	611,174	475x
*Q. dolicholepis*	Yichang, Hubei, China	KU240010	209,578	163x

### Repeat Elements Analysis

Tandem repeat sequences (>10 bp in length) were detected using the online program Tandem Repeats Finder (Benson, 1999), with 2, 7, and 7 set for the alignment parameters match, mismatch, and indel, respectively. The minimum alignment score and maximum period size were 80 and 500, respectively. REPuter (Kurtz et al., 2001) was used to find dispersed and palindromic repeats in which the minimal repeat size was 30 bp and the two repeat copies had at least 90% similarity. The gap size between palindromic repeats had a maximum length of 3 kb. All of the repeats found were manually verified and redundant results were removed. The positions and types of simple sequence repeats (SSRs) were ascertained using msatcommander (Faircloth, 2008). The minimum numbers of repeats were 10, 5, 4, 3, 3, and 3 for mono-, di-, tri-, tetra-, penta-, and hexanucleotides, respectively.

### Sequence Divergence Analysis

The alignments of the five complete chloroplast genome sequences were visualized using mVISTA (Frazer et al., 2004) in order to show interspecific variation. The percentage of variable characters for each coding and non-coding region with an aligned length of more than 200 bp was obtained based on the method of Zhang et al. (2011). Variable sites and parsimony-informative sites across the complete chloroplast genomes and LSC, SSC, and IR regions of the five taxa were calculated using DnaSP v5.0 (Librado and Rozas, 2009). Nucleotide substitutions were counted using MEGA 5.0 (Tamura et al., 2011), and indels were manually detected across the cp genomes. Selective pressures were computed for protein-encoding genes that were located in SC regions and one IR region. Non-synonymous (*K*_A_) and synonymous (*K*_S_) substitution rates were calculated using PAML with the yn00 program (Yang, 2007). There were 10 pairwise alignments for each gene, which contributed to a total of 790 *K*_A_/*K*_S_ values.

### Phylogenetic Analysis

Phylogenetic analysis was conducted based on 10 taxa, including five species in the current study, three other *Quercus* species (*Q. rubra*, *Q. spinosa*, and *Q. aquifolioides*), and two Fagaceae species (*C. mollissima* and *Castanopsis echidnocarpa*) that were used as outgroups. The sequences were aligned using MAFFT (Katoh and Standley, 2013) in GENEIOUS R8 with the default parameters set, and were manually adjusted in MEGA 5.0. Because molecular evolutionary rates differ in different cp genome regions, we constructed the phylogenetic tree using the following datasets: (1) the LSC region; (2) the SSC region; (3) the inverted repeat B (IRB) region; (4) the LSC + SSC regions; (5) the LSC + SSC + IRB regions; and (6) the complete chloroplast genome sequences.

Modeltest 3.7 (Posada and Crandall, 1998) was used to determine the best-fitting model for each dataset based on the Akaike information criterion. Maximum likelihood analysis was performed using RAxML v7.2.8 (Stamatakis, 2006) with 1000 bootstrap replicates. Bayesian inference was performed using the program MrBayes v3.1.2 (Ronquist and Huelsenbeck, 2003). Markov chain Monte Carlo simulations were independently run twice for 2 million generations, and sampling trees every 100 generations. Convergence was determined by examining the average standard deviation of split frequencies (<0.01). The first 25% of trees was discarded as burn-in, and the remaining trees were used to build a majority-rule consensus tree.

## Results

### Complete Chloroplast Genomes of Quercus Species

**FIGURE 1:**
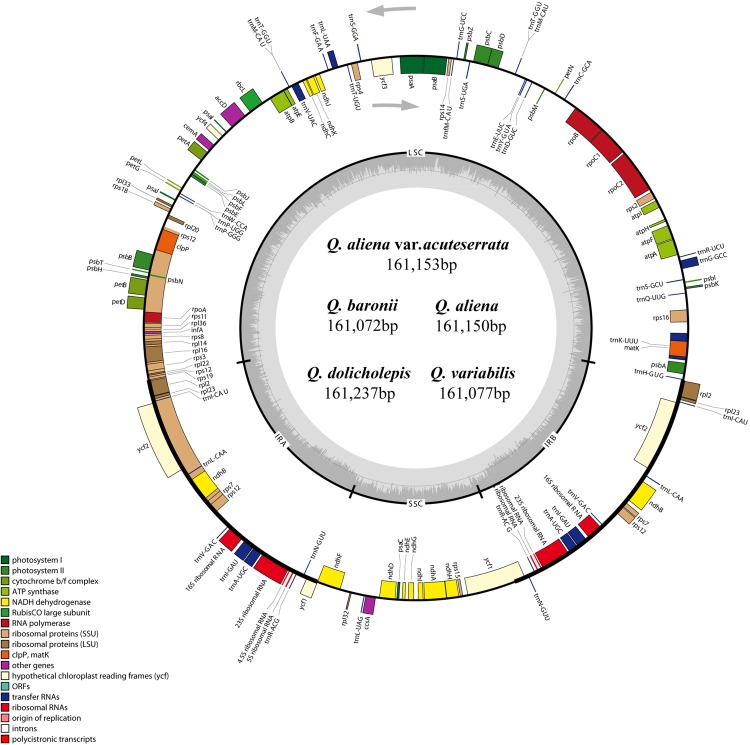
**Gene map of the five *Quercus* chloroplast genomes**. The genes shown outside of the circle are transcribed clockwise, while those inside are transcribed counterclockwise. Genes belonging to different functional groups are color coded. Dashed area in the inner circle indicates the GC content of the chloroplast genome.

**FIGURE 2:**
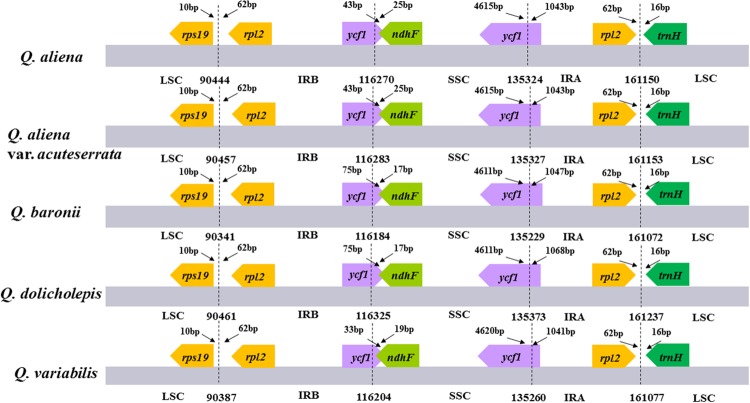
**The comparison of the LSC, IR, and SSC border regions among the five *Quercus* chloroplast genomes**. Number above the gene features means the distance between the ends of genes and the borders sites. These features are not to scale.

The five chloroplast genomes ranged in size from 161,072 bp (*Q. baronii*) to 161,237 bp (*Q. dolicholepis*; **Figure 1**). All of them displayed a typical quadripartite structure, and the same regions were of similar lengths (**Table 2**). Gene content and order were identical in the five species, and were similar to other published chloroplast genomes in Fagaceae (Jansen et al., 2011; Alexander and Woeste, 2014; Dane et al., 2015; Du et al., 2015; Lu et al., 2015). Although genomic structure and size were highly conserved in the five cp genomes, the IR/SC boundary regions still varied slightly (**Figure 2**). For example, the distance from the *ycf1* 5′ end to the junction of IRB/SSC was 43 bp in *Q. aliena* and *Q. aliena* var. *acuteserrata*, 75 bp in *Q. baronii* and *Q. dolicholepis*, and 33 bp in *Q. variabilis*. The assembled cp genomes encoded 134 genes, which consisted of 86 protein-coding genes, 40 transfer RNA (tRNA) genes, and 8 ribosomal RNA (rRNA) genes. Eighteen genes were duplicated in the IR region, including seven protein-coding genes, seven tRNA genes, and four rRNA genes (**Table 2**). A total of 14 protein-coding genes and 8 tRNA genes contained one or more introns (Supplementary Table S2). The GC content of each analyzed species was very similar in the same region or complete cp genome, but in the IR region it was clearly higher than in the other regions, possibly because of the high GC content of the rRNA (55.5%) that was located in the IR regions (**Table 2**).

**Table 2:** Characteristics of *Quercus* chloroplast genomes.

	*Q. baronii*	*Q. aliena*	*Q. aliena* var. *acuteserrata*	*Q. variabilis*	*Q. dolicholepis*
Size (bp)	161,072	161,150	161,153	161,077	161,237
LSC (bp)	90,341	90,444	90,457	90,387	90,461
SSC (bp)	19,045	19,054	19,044	19,056	19,048
IR (bp)	51,686	51,652	51,652	51,634	51,728
Protein-coding regions (bp)	80,250	80,121	80,073	80,133	80,322
Number of total genes	134	134	134	134	134
Number of protein-coding genes	86 (7)	86 (7)	86 (7)	86 (7)	86 (7)
Number of tRNA genes	40 (7)	40 (7)	40 (7)	40 (7)	40 (7)
Number of rRNA genes	8 (4)	8 (4)	8 (4)	8 (4)	8 (4)
Overall GC content (%)	36.81	36.83	36.83	36.78	36.80
GC content in LSC (%)	34.67	34.69	34.69	34.63	34.67
GC content in SSC (%)	30.88	30.90	30.92	30.83	30.86
GC content in IR (%)	42.73	42.77	42.77	42.77	42.74

*The numbers in brackets indicate the genes duplicated in the IR regions*.

### Repeat Elements Analysis

**FIGURE 3:**
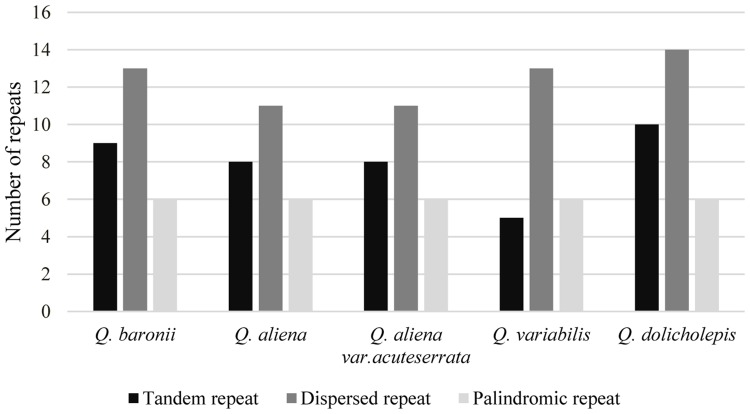
**Repeat number**. Histogram showing the number of three repeat types in five *Quercus* complete cp genomes.

The numbers and distributions of all of the repeat types in the five cp genomes were similar and conserved (**Figure 3**). There were 132 repeats, which included tandem, dispersed, and palindromic repeats. The lengths of the repeat units ranged from 14 to 40 bp. Most of them were distributed in intergenic or intron regions, and only a minority were located in gene regions (*ycf1*, *ycf2*, *psaA*, *psaB*, *trnS*-*GCU*, *trnS*-*UGA*, *trnG*-*GCC*, *trnG*-*UCC*, *trnS-UGA*, and *trnS-GGA*; Supplementary Table S3). We then analyzed the cp genome SSRs, which are often used as genetic markers in population genetics and evolutionary studies. The most abundant were mononucleotide repeats, which accounted for about 80% of the total SSRs, followed by dinucleotides (**Table 3**). Overall, there were slightly more tetranucleotide repeats than trinucleotide repeats, and penta- and hexanucleotides were very rare across the cp genomes. Protein-coding regions accounted for approximately half of the lengths of the cp genomes but only contained about 13% of the total SSRs, which meant that the SSR distribution was uneven across the cp genomes. SSRs located in the coding DNA sequence (CDS) region were mainly found in *rpoC2* and *ycf1*; *rpoB*, *atpB*, *accD*, *ndhF*, *rpl32*, and *ndhD* contained the remaining SSRs (Supplementary Table S4).

**Table 3:** Types and number of SSRs in cp genomes.

	*Q. baronii*	*Q. aliena*	*Q. aliena* var. *acuteserrata*	*Q. variabilis*	*Q. dolicholepis*
Mononucleotide repeats	84	85	86	87	85
Dinucleotide repeats	14	12	12	15	14
Trinucleotide repeats	4	3	3	3	3
Tetranucleotide repeats	6	3	4	6	6
Pentanucleotide repeats	2	2	1	1	2
Hexanucleotide repeats	1	0	0	0	1
All types in complete cp genome	111	105	106	112	111
All types in protein-coding regions	14	14	14	13	14

### Sequence Divergence Analysis

**FIGURE 4:**
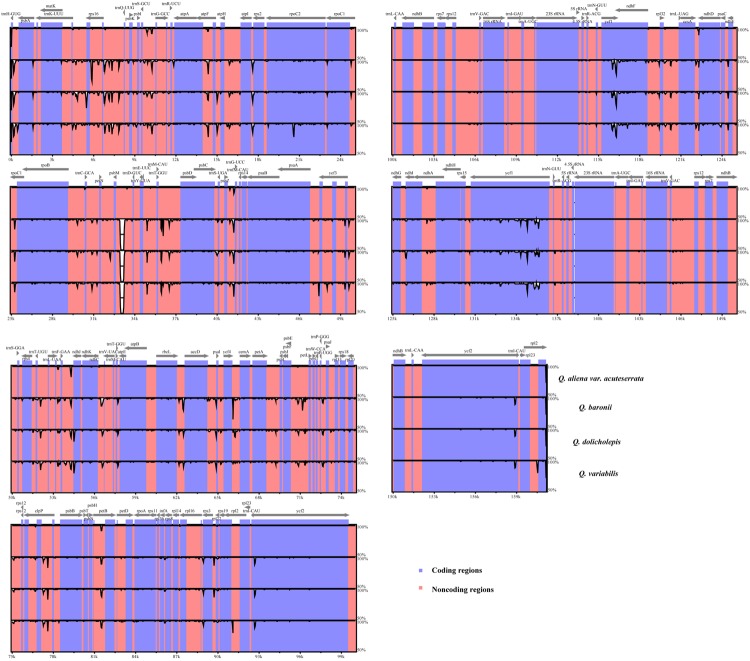
**Sequence identity plot comparing the five *Quercus* chloroplast genomes with *Q. aliena* as a reference by using mVISTA**. The *y*-axis represents % identity ranging from 50 to 100%. Coding and non-coding regions are marked in purple and pink, respectively.

**FIGURE 5:**
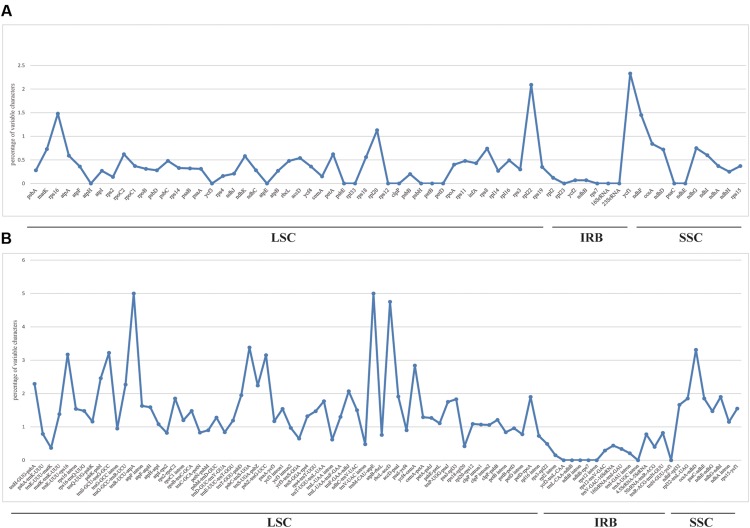
**Percentage of variable characters in aligned five *Quercus* chloroplast genomes**. **(A)** Coding region. **(B)** Non-coding region. These regions are oriented according to their locations in the chloroplast genome.

We used mVISTA to perform a sequence identity analysis, with *Q. aliena* as a reference (**Figure 4**). The alignment revealed high sequence similarity across the five cp genomes, which suggests that they are highly conserved. As expected, non-coding and SC regions exhibited higher divergence levels than coding and IR regions, respectively. The percentage of variation in non-coding regions ranged from 0 to 5.00%, with an average of 1.38%, which was threefold higher than that in the coding regions (0.40% on average; **Figure 5**). In the non-coding regions, the mean percentages of variations in the LSC, SSC, and IR regions were 1.59, 1.87, and 0.23%, respectively, which demonstrated that the IR region had fewer mutations and was highly conserved. However, in the coding regions, there were no significant differences among the regions (0.38, 0.54, and 0.32% for LSC, SSC, and IR regions, respectively), because there was a highly variable gene, *ycf1* (2.33%), that was located in the IR region. Genes that were located in SC regions (*rps16*, *rpl20*, *rpl22*, and *ndhF*) also exhibited higher variability (average value > 1%) than the other genes.

We then investigated sequence divergence patterns in the five cp genomes. We found 904 single nucleotide variants (SNVs; 0.56%) across the complete cp genomes of the five taxa, including 620 parsimony-informative sites (0.38%). There was a relatively small number of SNVs in the IR regions and coding sequences (Supplementary Table S5). The numbers of nucleotide substitutions and indels varied from 16 to 720 and 4 to 108, respectively (**Table 4**). There were always fewer transitions than transversions, and there were no transitions between *Q. aliena* and *Q.* aliena var. *acuteserrata*. Among the substitution events in the CDS region, all of the pairwise sequence comparisons showed that there was almost an equal number of synonymous and non-synonymous substitutions (**Table 5**); however, several NADH genes had more non-synonymous than synonymous substitutions, and most photosynthetic genes had only a few non-synonymous substitutions (Supplementary Tables S6 and S7). Most indels were located in non-coding regions, but some were also detected in *psbA*, *rpoC2*, *rpl22*, *ycf1*, *ycf2*, and *ndhF*. Interestingly, *ycf1* had the most number of indels (Supplementary Table S8).

**Table 4:** Numbers of nucleotide substitutions and indels in five complete cp genomes.

	*Q. baronii*	*Q. aliena*	*Q. aliena* var. *acuteserrata*	*Q. variabilis*	*Q. dolicholepis*
*Q. baronii*	/	716 (337/379)	715 (339/376)	245 (109/136)	190 (77/113)
*Q. aliena*	98	/	16 (0/16)	720 (351/369)	677 (326/351)
*Q. aliena* var. *acuteserrata*	97	4	/	719 (353/366)	680 (328/352)
*Q. variabilis*	46	108	107	/	222 (106/116)
*Q. dolicholepis*	26	102	99	43	/

*The upper triangle shows the number of nucleotide substitutions in complete cp genomes, the number of Ts and Tv are given in brackets. The lower triangle indicates the number of indels*.

**Table 5:** Numbers of synonymous and non-synonymous substitutions in CDS and ratios of Ts/Tv in complete cp genomes.

	*Q. baronii*	*Q. aliena*	*Q. aliena* var. *acuteserrata*	*Q. variabilis*	*Q. dolicholepis*
*Q. baronii*	/	0.89	0.90	0.80	0.68
*Q. aliena*	111/117	/	0	0.95	0.93
*Q. aliena* var. *acuteserrata*	110/117	1/2	/	0.96	0.93
*Q. variabilis*	44/46	120/125	119/126	/	0.91
*Q. dolicholepis*	25/28	113/112	112/113	47/42	/

*The upper triangle shows the ratios of Ts/Tv of complete cp genomes. The lower triangle indicates the number of synonymous and non-synonymous substitutions in CDS*.

To estimate selection pressures, ratios of non-synonymous (*K*_A_) versus synonymous (*K*_S_) substitutions were calculated for 79 protein-coding genes (Supplementary Table S9), and 293 pairwise comparison results were obtained. The *K*_A_/*K*_S_ ratios of the remaining comparisons could not be calculated due to *K*_S_ = 0. Four genes (*ndhA*, *ndhK*, *petA*, and *ycf1*) had *K*_A_/*K*_S_ ratios above 1.0, indicating that these genes are under positive selection.

### Phylogenetic Analysis

**FIGURE 6:**
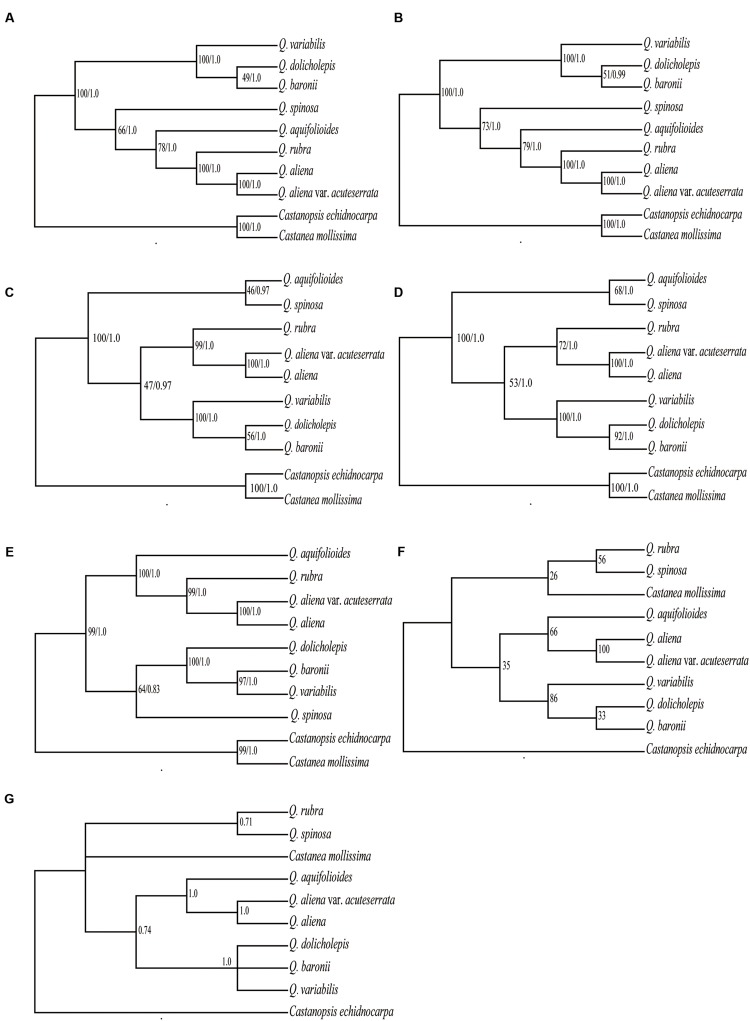
**Phylogeny of the 10 Fagaceae species inferred from ML and BI analyses of different data partitions**. **(A)** Whole chloroplast genome. **(B)** LSC+SSC+IRB region. **(C)** LSC+SSC region. **(D)** LSC region. **(E)** SSC region. **(F)** ML topology of IRB region. **(G)** BI topology of IRB region. The numbers associated with each node are bootstrap support values and posterior probability values in **(A–E)**. The numbers associated with each node are bootstrap support values and posterior probability values in **(F,G)**, respectively.

Six data partitions (**Table 6**) from the 10 Fagaceae cp genomes were used to construct the phylogenetic trees (**Figure 6**). All of the six datasets produced similar phylogenetic trees with moderate-to-high support, except for the IRB dataset, which received poor support. All of the datasets indicated that *Q. aliena* and *Q. aliena* var. *acuteserrata* form a monophyletic clade and then cluster with *Q. rubra*. Another monophyletic branch showed that *Q. baronii* appeared to be more closely related to *Q. dolicholepis* than to *Q. variabilis*. Differences in topological structure mainly involved the placements of *Q. spinosa* and *Q. aquifolioides*, which belong to Sect. *Brachylepides*. For example, in datasets 5 (LSC + SSC + IRB regions) and 6 (complete cp genome sequences), they clustered with *Q. rubra*; however, in datasets 1 (LSC region) and 4 (LSC + SSC regions), they formed a monophyletic group.

**Table 6:** Model in ML and BI analysis.

	Best fit model	Model in ML	Model in BI
LSC region	TVM+I+G	GTR+G	TVM+I+G
SSC region	TVM+I+G	GTR+G	TVM+I+G
IRB region	K81uf+I	GTR+G	K81uf+I
LSC + SSC region	TVM+I+G	GTR+G	TVM+I+G
LSC + SSC + IRB region	K81uf+I +G	GTR+G	K81uf+I +G
Complete cp genome	K81uf+I +G	GTR+G	K81uf+I +G

## Discussion

### Chloroplast Sequence Evolution

Although cp genomes are highly conserved in terms of genomic structure and size, the IR/SC junction position change may be caused by the contraction or expansion of the IR region, which is a common evolutionary phenomenon in plants (Kim and Lee, 2004; Hansen et al., 2007; Wang et al., 2008; Huang et al., 2014).

Larger and more complex repeat sequences may play an important role in the rearrangement of cp genomes and sequence divergence (Timme et al., 2007; Weng et al., 2013); therefore, we investigated the numbers and distributions of tandem, dispersed, and palindromic repeats. We found that repeats in different species were usually located in the same genes (*ycf1* and *ycf2*), or genes with similar functions (*psaB*/*psaA*, *trnS*-*GCU/trnS*-*UGA*, *trnG-GCC/trnG-UCC*, and *trnS-UGA/trnS-GGA).*

Understanding nucleotide substitution rates is of fundamental importance in molecular evolution (Muse and Gaut, 1994), and indels play a significant role in evolutionary processes (Britten et al., 2003). Based on the numbers and distribution of SNVs, indels, and proportions of variability, the IR regions were more conserved than the SC regions. During the process of searching for SNVs and indels, we found that the cp genome sequences of *Q. baronii*, *Q. dolicholepis*, and *Q. variabilis* had similar mutation modes, while the other two species shared another mutation mode. Therefore, the phylogenetic relationships of these species may be affected by different mutation modes. Transitions occur at higher frequencies than transversions in almost all DNA sequences, and transition/transversion bias is a general property of DNA sequence evolution (Yang and Yoder, 1999). However, all of the pairwise sequence comparisons in our study revealed that there was a greater number of transversions than transitions. This has also been found in other taxa (Cai et al., 2015; Song et al., 2015; Kong and Yang, 2016), and may be due to a high AT content in the cp genome; transversion substitutions usually occur in datasets with a high AT content (Morton and Clegg, 1995; Morton et al., 1997). This bias may also be associated with genome content and the genetic characteristics of codons (Yang and Yoder, 1999; Morton, 2003). The estimation of synonymous and non-synonymous substitution rates may play an important role in understanding the dynamics of molecular evolution, and non-synonymous substitutions could be subject to natural selection during the evolutionary process (Yang and Nielsen, 2000; Seo and Kishino, 2008). In this study, the numbers of synonymous and non-synonymous substitutions in the CDS regions were almost equal. However, in several NADH genes, more non-synonymous substitutions than synonymous substitutions were detected, while most photosynthetic genes had only a few non-synonymous substitutions, possibly due to strong selection pressure during cp genome evolution (Matsuoka et al., 2002).

Because of complex evolutionary issues in *Quercus*, its taxonomy is still difficult to assess. Barcoding is a molecular tool that is used to identify living organisms (Hebert et al., 2003). The loci *rbcL*, *matK*, and *trnH*/*psbA*, and nuclear ribosomal internal transcribed spacers, are recommended regions for DNA barcoding in plants (CBOL Plant Working Group, 2009; Hollingsworth et al., 2011). In a DNA barcoding study of *Quercus*, these plastid markers and an extra locus (*rpoC1*) had extremely low discriminatory power (Piredda et al., 2011; Simeone et al., 2013). Therefore, we chose the five most variable coding regions and 14 most variable non-coding regions that might be regarded as potential molecular markers for *Quercus* species, with variation percentages that exceeded 1 and 2%, respectively. They were *rps16*, *rpl20*, *rpl22*, *ycf1*, *ndhF*, *trnH*-*GUG*/*psbA*, *trnK*-*UUU*/*rps16*, *psbK*/*psbI*, *trnS*-*GCU*/*trnG*-*GCC*, *trnG*-*GCC*/*trnR*-*UCU*, *trnR*-*UCU*/*atpA*, *psbC*/*trnS*-*UGA*, *trnS*-*UGA*/*psbZ*, *psbZ*/*trnG*-*UCC*, *trnF*-*GAA*/*ndhJ*, *trnM*-*CAU*/*atpE*, *rbcL*/*accD*, *ycf4*/*cemA*, and *ccsA*/*ndhD*. Primers for these regions are shown in Supplementary Table S10. Further work is still necessary to determine whether these highly variable regions could be used in *Quercus* phylogenetic analyses, or serve as candidate DNA barcodes.

Our analysis indicated that four genes were under positive selection (*ndhA*, *ndhK*, *petA*, and *ycf1*). Eleven genes (*ndhA–ndhK*) are found in the cp genomes of most land plants, and encode a NAD(P)H dehydrogenase (NDH) complex that is involved in photosystem I cyclic electron transport and chlororespiration (Kofer et al., 1998; Shikanai et al., 1998). The chloroplast NDH complex is divided into A, B, and membrane and lumen subcomplexes; *ndhA* is a member of a membrane subunit and *ndhK* belongs to subcomplex A (Peng et al., 2011). The chloroplast NDH monomer, which is sensitive to strong light intensity, might have changed drastically to develop novel functions for stress resistance (Peng et al., 2011). *petA* encodes the apoprotein of cytochrome *f*, which is a membrane component of the cytochrome *bf* complex and has the function of transferring electrons (Gray, 1992). *ycf1* is one of the largest plastid genes, and encodes a protein that is a component of the chloroplast inner envelope membrane protein translocon (Kikuchi et al., 2013). Although this gene appears to be essential for cell survival in tobacco (Drescher et al., 2000), it is a pseudogene or has been lost in various groups, such as rice, maize, palm, and some Geraniaceae species (Maier et al., 1995; Yang et al., 2010; Weng et al., 2013). It has also been shown to be subject to positive selection in many lineages (Greiner et al., 2008; Carbonell-Caballero et al., 2015; Hu et al., 2015).

### Phylogenetic Analysis

The phylogenetic trees, which were based on different datasets, produced similar topological structures except for the IRB dataset, possibly because IRB is more conserved and provides fewer variable sites than SC regions. *Q. aliena* and *Q. aliena* var. *acuteserrata*, which belong to Sect. *Quercus*, had the closest relationship among the species, because *Q. aliena* var. *acuteserrata* is considered a variant of *Q. aliena* (Wu et al., 1994). *Q. rubra*, which belongs to Sect. *Lobatae*, always had a close relationship with the above two species, and the phylogenetic trees also showed that Sect. *Lobatae* is a sister clade of Sect. *Quercus* (Hubert et al., 2014). *Q. dolicholepis* is closely related to *Q. baronii*, and both of them are members of Sect. *Echinolepides*, which is consistent with their morphological characteristics. A phylogenetic tree that was based on morphological characteristics showed that the two deciduous sections were closely related, and formed a sister clade to an intermediate group (Pu et al., 2001); however, the phylogeny inferred from the cp genomes showed that deciduous oaks should not be clustered in the same clade. *Q. variabilis*, which belongs to Sect. *Aegilops*, was clustered with an intermediate group. Furthermore, a phylogenetic reconstruction of 108 *Quercus* species (including *Q. variabilis*, *Q. aliena*, and *Q. dolicholepis* from China) based on multiple nuclear genes showed that *Q. variabilis* is more closely related to *Q. dolicholepis* than to *Q. aliena* (Hubert et al., 2014). Across the different datasets, the positions of *Q. spinosa* and *Q. aquifolioides* were not consistent, and the bootstrap values of the two species were not high enough (<80%), probably because the limited number of species in our study might have influenced the analysis. Therefore, it is necessary to use more species in order to verify the relationships among different sections. Overall, the phylogenetic relationships inferred from the cp genome data obtained high support values and were similar to those indicated by nuclear genes data, which suggests that cp genome data can effectively resolve the phylogenetic relationships of this genus.

## Author Contributions

All authors listed, have made substantial, direct and intellectual contribution to the work, and approved it for publication.

## Conflict of Interest Statement

The authors declare that the research was conducted in the absence of any commercial or financial relationships that could be construed as a potential conflict of interest.

## Footnotes

1 http://ogdraw.mpimp-golm.mpg.de/
